# Surface Acoustic Wave (SAW)-Enhanced Chemical Functionalization of Gold Films

**DOI:** 10.3390/s17112452

**Published:** 2017-10-26

**Authors:** Gina Greco, Matteo Agostini, Richie Shilton, Marco Travagliati, Giovanni Signore, Marco Cecchini

**Affiliations:** 1 National Enterprise for nanoScience and nanoTechnology (NEST), Istituto Nanoscienze-CNR and Scuola Normale Superiore, Piazza San Silvestro 12, 56127 Pisa, Italy; gina.greco@sns.it (G.G.); matteo.agostini@sns.it (M.A.); marco.travagliati@gmail.com (M.T.); giovanni.signore@sns.it (G.S.); 2 Center for Nanotechnology Innovation@NEST, Istituto Italiano di Tecnologia, Piazza San Silvestro 12, 56127 Pisa, Italy; richieshilton@gmail.com

**Keywords:** functionalization, microfluidics, surface acoustic waves (SAWs)

## Abstract

Surface chemical and biochemical functionalization is a fundamental process that is widely applied in many fields to add new functions, features, or capabilities to a material’s surface. Here, we demonstrate that surface acoustic waves (SAWs) can enhance the chemical functionalization of gold films. This is shown by using an integrated biochip composed by a microfluidic channel coupled to a surface plasmon resonance (SPR) readout system and by monitoring the adhesion of biotin-thiol on the gold SPR areas in different conditions. In the case of SAW-induced streaming, the functionalization efficiency is improved ≈5 times with respect to the case without SAWs. The technology here proposed can be easily applied to a wide variety of biological systems (e.g., proteins, nucleic acids) and devices (e.g., sensors, devices for cell cultures).

## 1. Introduction

Surface chemical and biochemical functionalization consists of adding new functions, features, or capabilities to a material surface by changing its chemistry. It is a fundamental technique that is widely applied in many fields, such as chemistry, materials science, biological engineering, textile engineering, and nanotechnology. Surface wettability [1,2], biocompatibility [3,4], and sensing capabilities [5,6] are some of the material properties that can be finely tuned by this process.

A variety of functionalization strategies for gold films (a very common surface coating for integrated chips) are currently available [7]. For example, self-assembled monolayers (SAMs) of alkanethiolates or disulfides have been widely exploited for sensing applications [8] and for improving surface antifouling [9,10,11]. Typically, the immobilization of molecules on gold is based on passive absorption driven by hydrophobic and electrostatic interactions [12], or on covalent coupling [13]. Similar strategies can be applied to other surface materials or for different applications. For example, SAMs of alkyltrichlorosilanes can be used to tune silica surface hydrophobicity [14] and SAMs of polythiophene conductive polymers can improve the biocompatibility and electrical impedance of neural electrodes [15].

The use of microfluidic systems is a strategy that can improve surface functionalization. Indeed, they are able to reduce reagent volumes and provide a high spatiotemporal control of the concentration profiles [16]. Microfluidic devices can also enhance analytical sensitivity, temperature control, portability, automatization, and parallelization [17,18,19,20,21,22]. The process of surface functionalization can be easily monitored in microfluidic systems by embedded surface plasmon resonance (SPR) sensors. SPR is a label-free optical detection method that exhibits a high sensitivity to surface modifications and it is fully compatible with integrated systems and real-time measurements [23,24,25,26,27]. The major drawback of microfluidic chips is that, owing to their small dimension, laminar flows dominate the fluid dynamics, leading to prohibitively long mixing times [28]. One strategy to overcome this issue is the exploitation of surface acoustic wave (SAW)-induced acoustic streaming. SAWs are mechanical oscillations which propagate along the surface of a crystal. They can be generated by means of interdigital transducers (IDT) patterned on piezoelectric materials. When a SAW impinges on a liquid, the acoustic energy diffracts into the liquid owing to the sound velocity mismatch between the substrate and the liquid, causing a longitudinal pressure wave to be generated. This wave gives rise to the acoustic streaming [28], a net fluid motion that can lead to fast mixing, which also can occur in the case of a low Reynolds number regime [29,30,31,32,33,34]. 

Here, by using an integrated SPR microfluidic sensor, we demonstrate that SAW-induced acoustic streaming can also improve surface functionalization efficiency. To the best of our knowledge, this is the first method to enhance functionalization that is not based on chemical approaches (usually achieved by engineering or redesigning the structure of the employed ligand). We measure chip functionalization upon SAW-streaming by monitoring the biotin-thiol (b-SH) adsorption on gold SPR areas coupled to a polydimethylsiloxane (PDMS) microchannel. In order to correct for parasitic heating effects, the chip temperature is characterized upon SAW activation. To this end, control SPR sensing areas that are not affected by acoustic streaming are included in the same chip.

## 2. Materials and Methods

### 2.1. Chip Design, Fabrication, and Assembly

**Figure 1:**
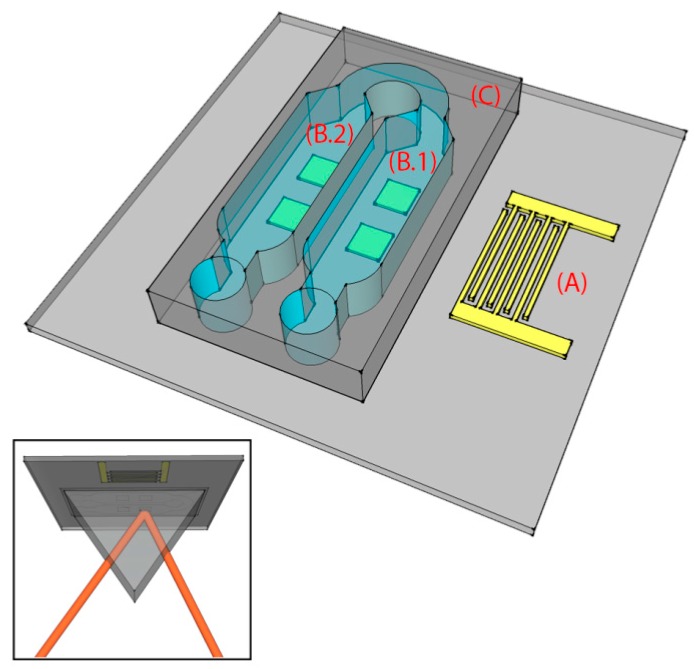
Schematic of the surface acoustic wave (SAW)-enhanced surface plasmon resonance (SPR) chip. The chip is characterized by three main parts, patterned on a lithium niobate (LN) substrate: an interdigital transducer (IDT) for SAW excitation (A), four SPR sensing areas (B), and a polydimethylsiloxane (PDMS) microchannel with two microchambers (C). The microchambers are designed so that the SAWs are present only in B.1, while they are totally damped before reaching B.2. The inset shows the chip (seen from below) in the Kretschmann configuration.

The chip consists of a lithium niobate (LN) substrate with four gold SPR sensing areas and a PDMS microchannel, as depicted in Figure 1. The 1 inch × 1 inch substrate consists of a 0.5-mm thick 128°Y-cut X-rotated LN crystal. On top of the LN substrate, a gold single-finger IDT (19 finger pairs, 50% metallization ratio) and four gold SPR sensing areas were fabricated along the X crystallographic direction, Figure 1. The IDT has 80 μm periodicity, corresponding to the SAW wavelength (λ), λ/4 fingers, and an acoustic aperture of 5 mm. The number of finger pairs was chosen to match the impedance at 50 Ω, whilst the LN substrate was chosen for its excellent electromechanical coupling coefficient for optimal SAW generation.

**Figure 2:**
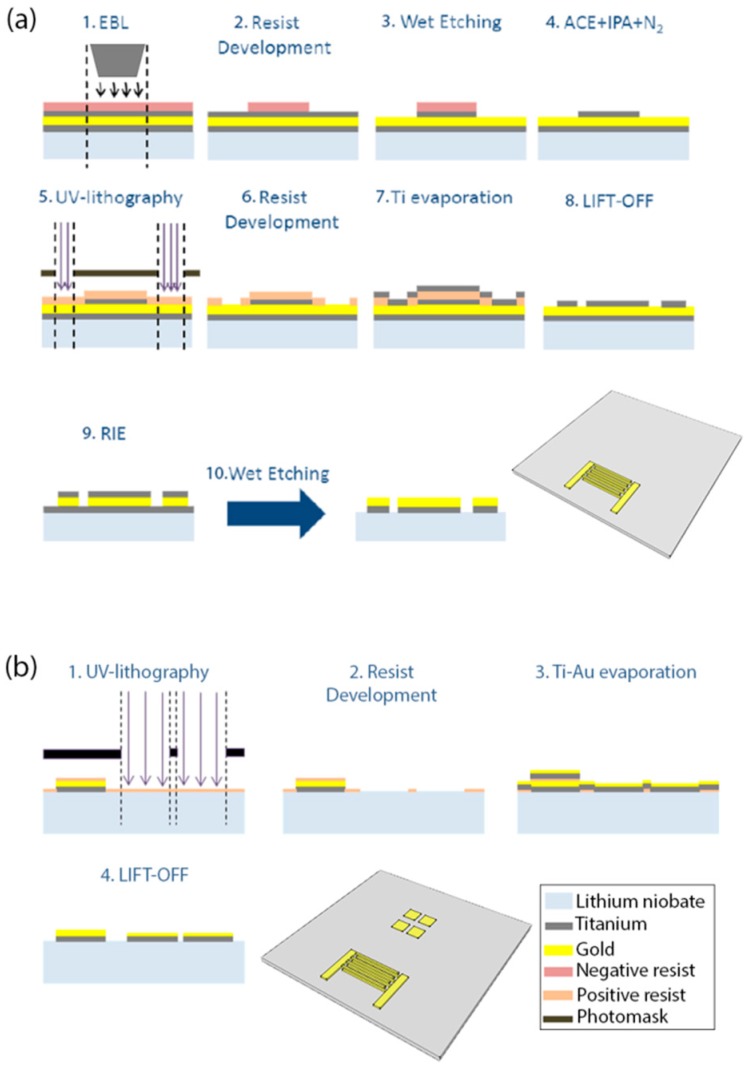
Fabrication process for the (**a**) IDT and (**b**) SPR sensing areas. (**a**) The first step is the electron beam lithography (EBL) of the IDT fingers (1–4). Next, the bus-bars of the IDT are patterned by UV-lithography followed by Ti evaporation and lift-off (5–8). Reactive ion etching (RIE) with Ar and wet etching with hydrofluoric acid (HF) are used to etch the Au and Ti, respectively, as last the step of the IDT fabrication. (**b**) UV-lithography (1–2) of four square areas is followed by the evaporation of Ti and Au (3). The last step is the lift-off (4) of the Ti-Au layer except for the four SPR sensing areas.

The fabrication procedure of the chip is shown schematically in Figure 2. It consists of the IDT (Figure 2a), sensing areas (Figure 2b), and microchannel fabrication. The substrate was cleaned with acetone (ACE) and isopropanol (IPA) (Sigma Aldrich, Italy, Milan), dried under nitrogen flux, and then exposed to plasma oxygen cleaning (0.2 mbar, 100 W, 1 min). The IDT was fabricated by depositing Ti-Au-Ti (10 nm, 100 nm, and 30 nm, respectively) on the LN surface. A layer of AR-300-80 (Allresist GmbH, Strausberg, Germany) was spin-coated at 4000 rpm for 1 min and soft baked for 5 min at 90 °C. Next, ma-N 2403 (Microchem) was spin-coated at 6000 rpm for 1 min and soft baked for 1 min at 90 °C. The fingers were patterned by electron beam lithography (EBL). The resist was developed in ma-D 525 (micro resist technology GmbH) for 1 min and the first layer of Ti was etched in a solution of hydrofluoric acid (HF), purchased by Sigma Aldrich, and deionized (DI) water (1:30 *v*/*v*). After cleaning, the chip was spin-coated with S1818 (Microchem) for 1 min at 6000 rpm, soft baked for 1 min at 90 °C, and UV lithography was used to pattern the bus-bars and the pads of the IDT. The resist was developed for 1 min in MF319 (Microchem). The 30-nm mask of Ti was deposited by thermal evaporation and the non-patterned metallized areas were removed by lift-off in ACE. Dry etching of Au was performed with reactive ion etching (RIE) and Ti was etched in the water-HF solution, as previously described. The four 4-mm^2^ square sensing areas were fabricated by lift-off, after UV-lithography and metal bilayer deposition (Ti-Au, 10-55 nm), as previously described for the fabrication of the bus-bars and pads.

**Figure 3:**
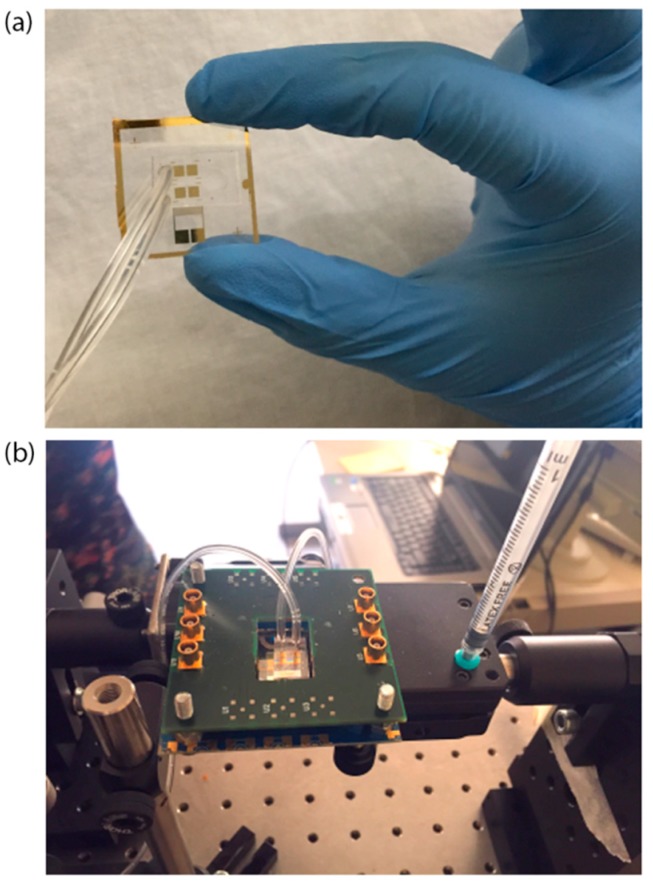
Picture of the sensor chip alone (**a**) and mounted on the holder with the printed circuit board (PCB) used to excite the SAWs (**b**) Tygon tubes are connected to a syringe (inlet) and to a waste beaker (outlet).

The PDMS (SYLGARD^®^ 184) microchannel was fabricated by replica molding and then covalently bonded on the chip. The mold consists of a silicon substrate where the microchannel (360 μm high) was patterned by UV exposure (15 mW/cm^2^ for 25 s) of the SU-8 2100 negative resist (spin-coated at 500 rpm for 15 s and at 800 rpm for 1 min, prebaked at 95 °C for 60 min and postbaked at 95 °C for 20 min). PDMS was chosen for its good optical and mechanical properties, tunable by changing the elastomer-curing agent volume ratio (1:10, in our case). The LN substrate and PDMS surface were activated using oxygen plasma at 100 W for 2 min and 10 W for 45 s, respectively. The two surfaces were aligned using an optical microscope, and finally baked for 1.5 h at 80 °C in an oven for complete irreversible bonding. The microchannel consisted of two microchambers (B.1 and B.2 in Figure 1) of a height of 360 μm and a width of 3 mm (0.5 mm larger, on each side, than the width of the square SPR sensing areas) that covered the four gold SPR surfaces (two in each chamber). Polytetrafluoroethylene (PTFE) tubes (Masterflex Tygon E-3603, Cole Parmer, Vernon Hills, IL, USA) with stainless steel tubing (200.010-A, Unimed S.A., Lausanne, Switzerland) and 2-mL syringes (Benefis) were used to inject liquids into the microchannel. The assembled chip is shown in Figure 3a,b.

### 2.2. SAW Excitation/Detection and Thermal Characterization

The IDT was preliminary tested by measuring the reflected power spectrum with a vector network analyzer (VNA), ENA Series Network Analyzer, E5071C, Agilent Technologies. The actual SAW resonance frequency was 48.1 MHz. The SAW amplitudes were measured with respect to the SAW excitation power by means of a laser Doppler vibrometer (LDV, UHF-120 Ultra High Frequency Vibrometer, Polytec, Mooresville, NC, USA). After characterization, SAWs were generated by applying a voltage sine wave at 48.1 MHz to the IDT at different powers using a radiofrequency (RF) generator (MXG Analog Signal Generator N5181A, Agilent Technologies, Santa Clara, CA, USA) connected to an amplifier (ZHL-5 W-1, MiniCircuits, Brooklyn, NY, USA). An infrared (IR) camera (FLIR A655sc) was used to measure and monitor the temperature of the LN substrate upon SAW activation until the equilibrium temperature was reached.

### 2.3. Fluid Dynamics Characterization

In order to visualize the fluid flow, we injected Milli-Q water containing 500-nm latex beads (L3280, Sigma-Aldrich) at a concentration of 7.6 × 10^8^ particles per mL and acquired 30 fps videos by using a brightfield inverted microscope (Eclipse TI, Nikon, Japan, Tokyo) equipped with a 4× objective and complementary metal oxide semiconductor (CMOS) camera (A602-f, Basler, Germany). We normalized the contrast of each frame, subtracted the time-averaged image to remove static objects, and superimposed all the frames, as in Reference [35]. To quantify the fluid velocity field, we analyzed the data with a micro particle image velocimetry (μPIV) code (Prana PIV, Virginia Polytechnic Institute and State University, Blacksburg, Virginia, USA). 

### 2.4. SPR Excitation/Detection Setup

**Figure 4:**
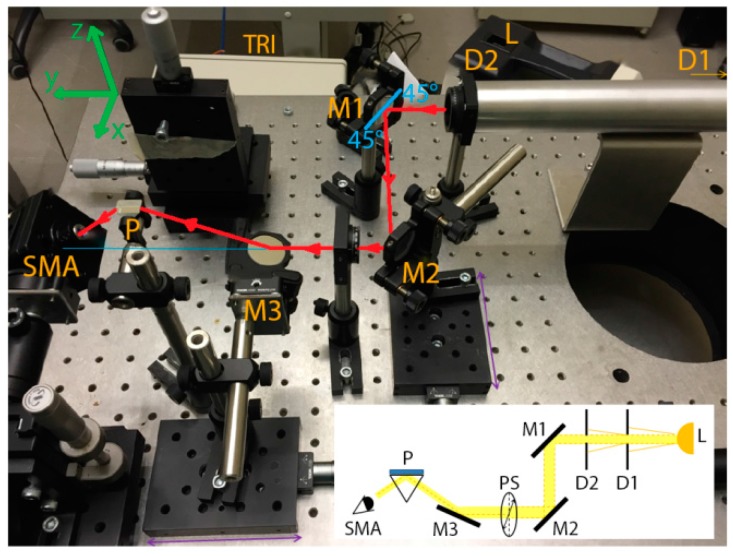
Picture of the optical setup for exciting and detecting SPRs. A schematic of the components of the optical setup is shown in the inset (bottom right). Polychromatic light (L) reaches the polarizer (PS) after passing through two diaphragms (D1 and D2) and two mirrors (M1 and M2). By means of a third mirror (M3) and a prism (P), the polarized light reaches the sensor (mounted with a holder on top of the prism) and excites the surface plasmons (SPs). SPR is detected with a spectrometer (SMA) which collects the polarized light reflected from the sensor.

A custom optical setup (shown in Figure 4) was used for the SPR generation in the Kretschmann configuration with wavelength modulation. A white light source (Leica CLS 150 XE) was used at its maximum power (150 W) to excite the surface plasmons (SPs). An uncoated SF11 10-mm micro right-angle prism (n_d_ = 1.7847), purchased from Edmund Optics, was used to couple the light at the fixed optimal angle of 28° with respect to the light beam direction. The SPR was detected by acquiring the reflectance spectra of p-polarized (which can excite SPs) and s-polarized (which cannot excite SPs) light with a TRISTAN light desktop spectrometer (MUT) working in the 400−800 nm range. SPR was detected as a dip in the normalized reflectivity:

$$R=\frac{I_{r,p}}{I_{r,s}}$$

where I_r_,_p_ is the intensity of the p-polarized light reflected by the gold area and I_r_,_s_ is the intensity of the reflected s-polarized light. As shown in Figure 4, the incident light beam passed through two diaphragms (D1 and D2) set at 1 mm and separated by 30 cm. The two diaphragms were connected by a metallic tube to minimize the light noise from the laboratory. Then, the light beam hit two silver mirrors (M1 and M2) placed at 45° with respect to the light beam direction. A linear polarizer (PS) in a rotation mount was placed between M2 and a third mirror (M3). A subminiature version A (SMA) optical fiber connector (SMA in Figure 4) was used to detect the reflectance spectra with the spectrometer. The optical fiber connector consisted of a lens with a central focal length of 18.24 mm, an alignment wavelength of 633 nm, and a numerical aperture (NA) of 0.15. All the components of the optical setup (where not specified) were purchased from Thorlabs, Inc.

### 2.5. Functionalization Protocols

b-SH was synthesized according to a reported procedure [36]. b-SH solutions were prepared by dissolving 1 mg/mL in water:ethanol (Sigma Aldrich, HPLC grade, 96%) 10:1 *v*/*v*. For the functionalization experiments, a water:ethanol (10:1 *v*/*v*) solution was injected into the microchannel using the disposable sterilized 2-mL syringe. Next, SAWs were turned on and we waited for 20 min until the device, with the solution injected, reached thermal equilibrium. Afterwards, measurements were taken from the SPR sensing areas (as shown in the inset of Figure 1) in the microchambers B.1 (where streaming and heating effects induced by the SAWs were present) and B.2 (where only the heating induced by the SAWs was present). Air was then injected to empty the microchannel and to inject the b-SH solution for the functionalization of the gold SPR sensing areas. After 20 min of thermalization, measurements were taken in B.1 and B.2. In the case without SAWs the experimental protocol was identical, with the exception that the SAWs were switched off.

## 3. Results and Discussion

### 3.1. Chip Fluid Dynamics Characterization

**Figure 5:**
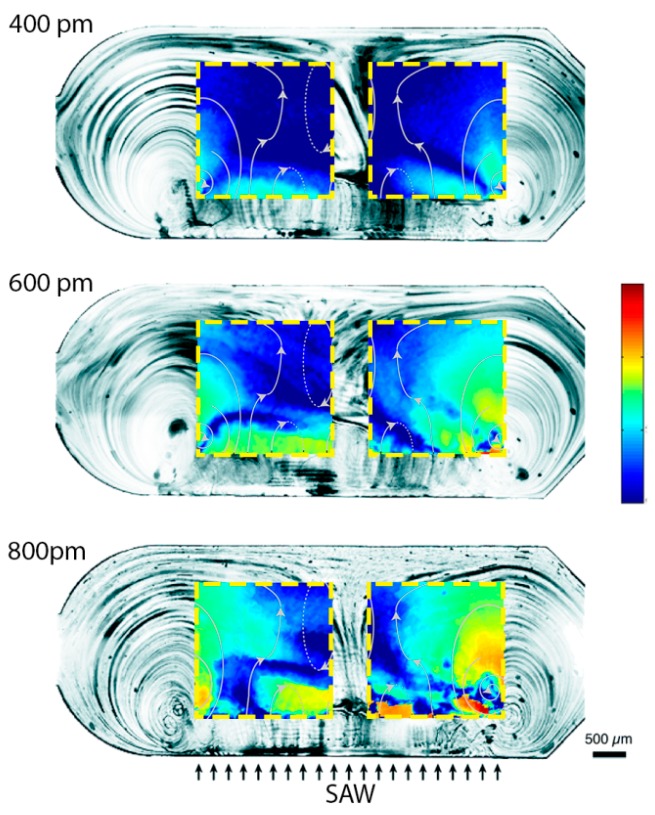
SAW-induced streaming characterization for different wave amplitudes. The streamlines of the fluid flow in the microchannel are shown for three different powers. The micro particle image velocimetry (μPIV) velocity fields on the two gold SPR areas are superimposed to the streamlines. The color bar represents the logarithm of the velocity and ranges from 10 μm s^−1^ to 1800 μm s^−1^.

Our chip was designed such that the SAWs were completely damped before the second microchamber (B.2 in Figure 1). Indeed, the microchambers were 3 mm wide and 1 mm distant from each other. Since for SAWs propagating on LN the damping length is 463 μm in water [37], SAW-induced streaming was only possible in the first chamber (B.1 in Figure 1), whereas in the second chamber (B.2 in Figure 1) only the heating effect of SAWs on the substrate was present. Among the different amplitudes exploited (Figure 5 shows the results obtained in the case of 400, 600 and 800 pm), SAWs with 800 pm amplitudes were chosen as optimal for inducing streaming, and used for all the successive experiments reported in this paper. At this amplitude, the heating of the substrate (as it will be shown in the next section) was tolerable for the chip and for the b-SH-gold binding. The streaming induces a complex streamline pattern (qualitatively similar in all the cases, as shown in Figure 5) which allows mixing inside the microchamber at velocities much higher than the ones typical of diffusion only. The fluid reached a maximum velocity of 1530 ± 80 μm s^−1^ with an average value of 160 ± 40 μm s^−1^ in the gold SPR area regions (Figure 5). From the (micro particle image velocimetry) μPIV analysis, it was possible to identify two main lateral vortices, a central jetting zone, and two slower central back rolls.

### 3.2. Thermal Characterization

**Figure 6:**
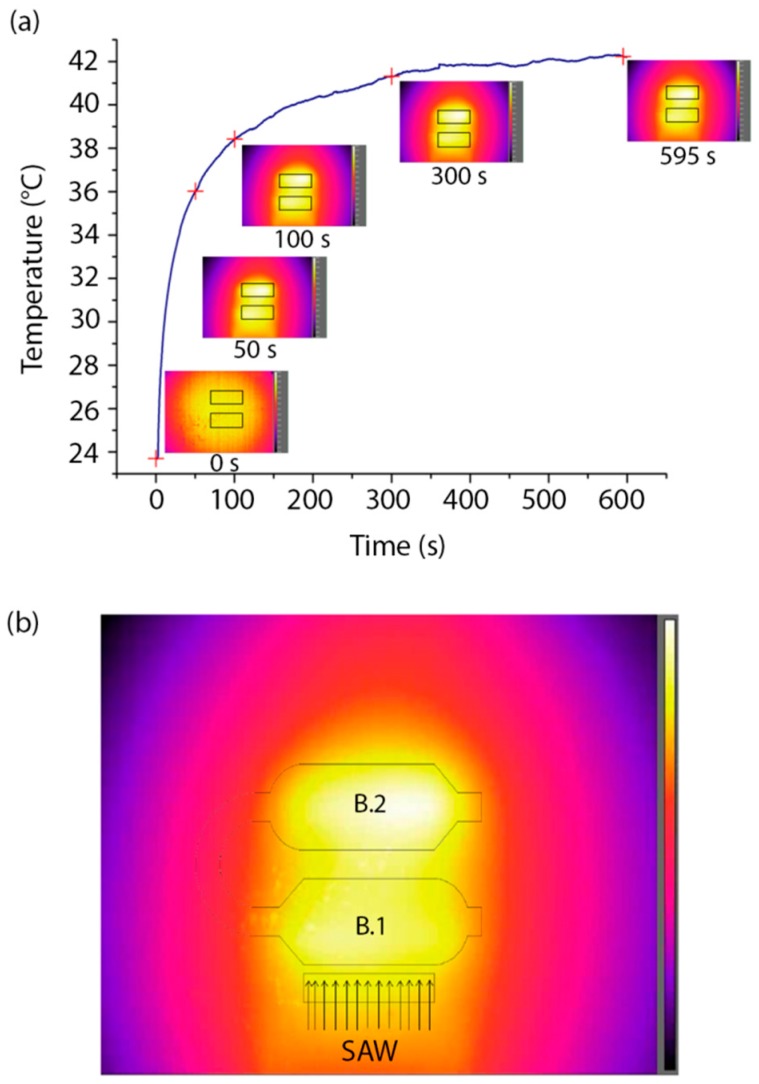
Thermal measurements in the presence of SAWs. (**a**) Heating of the LN substrate versus time (SAW activation at t = 0). The insets show five images from the thermal camera at t = 0 s, 50 s, 100 s, 300 s and 595 s, with the regions of interest (ROIs) used for calculating the average substrate temperature. (**b**) Temperature profile of the LN substrate at thermal equilibrium after SAW generation. A schematic of the microchannel is superimposed to the image. SAW direction from IDT is specified by the arrows. Color bar ranges from 28.8 °C to 42.0 °C.

The activation of SAWs also determines the heating of the sample, due to the RF excitation that induces Joule effect in the transducer [38] and SAW viscous dissipation into both the PDMS and liquid [39]. For this reason, we monitored the temperature of the chip while SAWs were active. The IR camera was used to measure the temperature of the piezoelectric substrate during the generation of SAWs until the equilibrium temperature was reached. In particular, we measured the temperature of the substrate in the region of the microchambers. The equilibrium temperature was reached after ≈8 min and was ≈17 °C higher than the starting temperature. Figure 6a shows the heating of the substrate following SAW activation (t = 0 s) until equilibrium. The temperature reported in this plot is the mean over the rectangular regions of interest (ROI) shown in the insets, which show images taken by the IR camera at t = 0 s, 50 s, 100 s, 300 s and 595 s from SAW activation. Figure 6b displays a representative temperature profile of the piezoelectric substrate at equilibrium.

Since the refractive index of a liquid depends on its temperature, the measurements of the SPR signal of the water-ethanol solution (b-SH reference solution) were taken at equilibrium (20 min after the injection of the solution). This precaution also allowed considering any change due to partial solvent evaporation or adsorption on PDMS that could affect the composition and hence the refractive index of the mixture.

### 3.3. Measurement of Functionalization Efficiency

**Figure 7:**
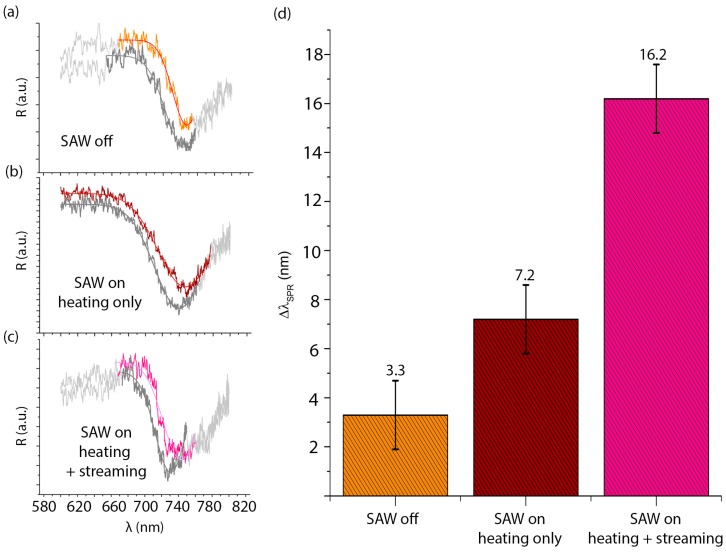
Representative SPR spectra in the case of water-ethanol (10:1 *v*/*v*; dark grey curves), and b-SH in water-ethanol (1 mg/mL) in the microchannel when SAWs are off (orange curve in (**a**), in the microchamber B.2; bordeaux curve in (**b**) where only the heating effect of the SAW is present; and magenta curve in (**c**) where both heating and streaming induced by the SAWs are present. (**d**) Average resonance wavelength shift in the case of b-SH in water-ethanol (1 mg/mL) to water-ethanol only without the SAW, with the heating effect only of the SAW and with the SAW, respectively.

We studied how SAW-induced streaming and heating affect the b-SH functionalization of the gold surfaces. b-SH in water-ethanol was used for the functionalization of the gold areas. Measurements were taken in the microchambers B.1 and B.2 with and without the presence of the SAWs. When the SAWs were active, measurements were made in the microchamber B.1 to evaluate the effect of acoustic streaming on functionalization, whereas we evaluated the heating effect alone by acquiring the same kind of data in the second microchamber B.2 (see schematic in Figure 1). SPR measurements were performed as described in Section 2.5. Measurements were taken up to 1 h to monitor the signal stability. Raw data of the wavelength spectrum of s- and p-polarized reflected light were processed to obtain the SPR curves for each configuration (SAW off, SAW on-streaming and heating, SAW on-heating only). For all cases, the resonance wavelengths were derived by a Gaussian fit (see Figure 7). 

The shifts of the resonance wavelength ($\Delta \lambda_{SPR}$), linearly proportional to the amount of b-SH bound on the gold SPR detection surfaces, are an indication of the efficiency of the surface functionalization. Their mean values, in different cases, are shown in Figure 7. The SAW-induced streaming yielded higher $\Delta \lambda_{SPR}$ with respect to the two control cases, suggesting a more efficient functionalization of the gold SPR areas. Indeed, $\Delta \lambda_{SPR}$ in the case with SAW was ≈5 times $\Delta \lambda_{SPR}$ in the case without SAW, while $\Delta \lambda_{SPR}$ in the heating-only area was only ≈2.2 times the no-SAW case. This can be explained since it is already known that the temperature increase enhances the thiol binding on gold, as shown in References [40,41]. However, the SAW-induced streaming improves the functionalization with respect to the non-turbulent microfluidic flows that would otherwise dominate, as in the cases without SAWs. A possible scenario might be that the SAW-induced streaming, other than improving recirculation and mixing through chaotic advection [42,43], enhances the probability of detachment of non-specifically bound species (i.e., b-SH interacting by hydrophobic interaction rather than by gold-thiol bonds) from the sensor surface, therefore increasing the probability of oriented b-SH-gold binding.

## 4. Conclusions

We demonstrated that SAWs can enhance the chemical functionalization of gold films. This has been shown by using a microfluidic system and an SPR readout system to quantitatively measure the amount of b-SH attached to gold areas. The SAW-induced effects of streaming and heating on the functionalization were also fully characterized, and their effect on functionalization was decoupled. The amount of bound b-SH on gold is ≈5 times higher in the case of SAW-induced streaming than in the case without SAWs, while in presence of heating alone it is ≈2 times higher. The chip that we showed in this article is, to the authors’ knowledge, the first attempt to mechanically improve chemical surface functionalization. The technology reported in this study can be further improved and applied to a great variety of biological systems (e.g., proteins, nucleic acids) and devices (e.g., sensors, devices for cell cultures).
